# What to do with non-responders to CGRP(r) monoclonal antibodies: switch to another or move to gepants?

**DOI:** 10.1186/s10194-023-01698-8

**Published:** 2023-12-05

**Authors:** Marta Waliszewska-Prosół, Doga Vuralli, Paolo Martelletti

**Affiliations:** 1 Department of Neurology, Wrocław Medical University, Borowska 213 Str, 50-556 Wrocław, Poland; 2 Department of Neurology and Algology, Neuroscience and Neurotechnology Center of Excellence (NÖROM), Faculty of Medicine, Neuropsychiatry Center, Gazi University, Ankara, Turkey; 3 School of Health Sciences, UnitelmaSapienza University of Rome, Rome, Italy

**Keywords:** CGRP, Gepants, Responders, Switching, Combination

## Abstract

In this editorial we aim to provide potential therapeutic options in patients who do not benefit from treatment with CGRP(r) monoclonal antibodies. Based on current real-life studies and analysis of practical and economic aspects, we will analyze the potential benefits of changing CGRP-targeted treatment.

New migraine-specific preventive therapies targeting the calcitonin gene-related peptide (CGRP) pathway are undoubtedly the biggest discovery in neurology in recent years. They have expanded the arsenal of anti-migraine drugs to include monoclonal antibodies (mAbs) targeting CGRP and orally administered small molecule CGRP receptor antagonists—gepants (atogepant and rimegepant) [1, 2].

Currently, monoclonal antibodies include erenumab, fremanezumab, galcanezumab and eptinezumab. Erenumab—the first antibody to be introduced into clinical practice—unlike the others, targets the CGRP receptor rather than the peptide itself [3]. Due to their long half-life, they can be used once a month. Fremanezumab can also be administered quarterly (in a triple dose) and the efficacy of therapy in both dosing regimens is comparable [4].

The efficacy of mAbs in the prevention of migraine attacks and their safety have been confirmed in randomized phase 3 clinical trials in patients with both episodic (EM) and chronic migraine (CM) [5]. In some real-world studies, non-responders are more numerous in CM than in EM which is most likely due to the fact that CM is not a homogeneous disease [6]. They have proven effective in resistant migraine patients with previous failures of prophylactic therapies and onabotulinum toxin A [7–9]. However, in clinical practice, treatment failures with these drugs are observed in up to 30–40% of patients [10].

## Switch to another mAbs

The question then arises whether, after such failure, it is worthwhile to include treatment with an antibody with a different mechanism of action especially considering the cost of treatment. And on the other hand, another important question in clinical practice is how long should we use one antibody before we find it ineffective? The rules regarding the length of prophylactic treatment are still contractual and, for many countries, individual and mainly limited by treatment costs and reimbursement [11]. Barbanti et al. showed that 55% of patients not responding to treatment in the first 3 months benefited from prolonged treatment and responded later—these are so-called *late responders* [12]. Therefore, it seems that the efficacy of anti-CGRP mAb should be evaluated after 6 months at the earliest.

Recent years bring many observations from actual medical practice and confirm that the lack of efficacy of one antibody does not exclude the possibility of a good effect after another.

There may be individual differences in response between MAbs. Switching between the two may therefore be appropriate in selected patients. Currently, there are no head-to-head studies directly comparing antibodies with each other. However, there are reports suggesting an advantage for antibodies interacting with a peptide compared to erenumab interacting with a receptor [13, 14].

Ziegler et al. showed that galcanezumab therapy proved beneficial in patients who had previously failed erenumab treatment [15]. In 2023, the FINESSE trial was published, which was designed to evaluate the efficacy of fremanezumab in patients with prior treatment failure with another antibody. In 153 patients with prior treatment failure (with erenumab or galcanezumab), switching to fremanezumab led to a ≥ 50% reduction in the number of days with migraine per month in 42.8% of patients [16]. Differences in antibody efficacy have been attributed to mechanisms of action, including effects on the blood–brain barrier. Functional magnetic resonance imaging studies showed different central responses depending on the antibody acting on the ligand and on the receptor. Galcanezumab reduced activity in the left thalamus, hypothalamus and bridge areas, while erenumab specifically reduced activation in the insula, thalamus, cerebellum and amygdala [17, 18]. Determinants of the response to a particular class of antibodies are still under investigation [19, 20]. In conclusion, there is currently insufficient evidence of the potential benefits of antibody switching and it seems rational to switch between different classes of antibodies, i.e. from erenumab, a CGRP receptor blocker, to a ligand-blocking mAb or vice versa and switching should be considered at the earliest after 6 months of treatment.

## Move to gepants

Gepants are CGRP-receptor antagonists. They are non-peptide small molecules that inhibit CGRP receptors, CGRP involved vasodilation and inflammation and trigeminovascular activation [21]. This group of drugs are developed for both acute migraine attack treatment and migraine prevention [22]. Rimegepant, ubrogepant and atogepant are the second generation oral gepants. Atogepant is exclusively developed for migraine preventive treatment while rimegepant has shown efficacy as both acute migraine medication and preventive treatment of episodic migraine. Zavegepant is the third generation gepant and its nasal spray was shown to be effective in acute treatment of migraine compared to placebo in a phase 3, double-blind, randomised controlled trial [23].

Rimegepant and atogepant are two gepants that can be use in the preventive treatment of episodic migraine. Atogepant is the only gepant that is also approved in the preventive treatment of chronic migraine. Rimegepant 75 mg every other day reduced the number of migraine days more than placebo in a phase 2/3 randomised double-blind placebo controlled trial and the rate of adverse events were similar between rimegepant and placebo [24]. Atogepant was shown to be effective in the preventive treatment of episodic migraine in phase 3 ADVANCE study with mean differences from placebo in the mean migraine days from baseline, -1.2 days for atogepant 10 mg, -1.4 days for atogepant 30 mg and -1.7 days for atogepant 60 mg (*p* < 0.001 for all comparisons) [25].

Gepants competitively inhibits CGRP receptor and rimegepant and atogepant are also antagonists of the AMY1 receptor which could be an advantage in patients who are unresponsive to ligand blocking mAbs [26, 27]. Elimination half-life of rimegepant and atogepant is approximately 11 h [28, 29]. The shorter half-lives of gepants compared to CGRP mAbs could be in favor of gepants for use in patients planning to conceive. Gepants could be eliminated rapidly from the body in case of pregnancy or any other urgent medical condition.

There have been no trials comparing the efficacy of CGRP mAbs and gepants for migraine prevention until recently. A recent double-blind, double-dummy, randomized controlled clinical trial evaluated whether galcanezumab, a CGRP mAb was superior to Rimegepant, a CGRP receptor antagonist, in the prevention of episodic migraine [30]. In this phase 4 study in which ≥ 50% reduction in monthly migraine headache days during the 3-month treatment period was the primary end-point, episodic migraine patients either received galcanezumab 120 mg subcutaneous (s.c.) injections monthly and an oral placebo tablet every other day (q.o.d.) or rimegepant 75 mg tablet q.o.d. and placebo s.c. per month [30]. Main secondary efficacy end-points were mean change from baseline in: 1) monthly migraine headache days across the 3 month treatment period and at months 3, 2 and 1, 2) monthly migraine days with acute migraine medication use across the 3 month treatment period, 3) Migraine-Specific Quality of Life Questionnaire (MSQ) version 2.1 Role Function-Restrictive (RF-R) domain at month 3 and 4) percentages of patients with ≥ 75% and ≥ 100% reduction in monthly migraine headache days across the 3 month treatment period [30]. Regarding the primary end-point, galcanezumab was not found superior to rimegepant and no statistical significance was reported regarding the secondary efficacy end-points. Across the 3-month treatment period, 62% of patients in galcanezumab group and 61% of rimegepant group had ≥ 50% response rate. The number of patients reporting treatment-emergent adverse events were similar between the two groups [30].

Rimegepant is the only gepant that was studied in relation to lactation. After a single dose rimegepant 75 mg administration to lactating healthy participants between 2 weeks-6 months postpartum, breast-milk was collected at 0, 1, 2, 4, 8, 12, 16, 24, 32 and 36 h post-dose and the relative infant dose (RID) was calculated. Breastfeeding is usually considered acceptable when RID is < 10% and RID for rimegepant 75 mg was found to be 0.51% [31]. Even though this result is in favor of rimegepant use during lactation, more studies are required.

A rapid onset of preventive effect was reported with atogepant. In a phase III trial (ADVANCE), atogepant was reported to be effective starting from the first day following treatment initiation and a sustained reduction in monthly migraine days was shown across the 3 month treatment period [32].

Safety profile of gepants are similar to mAbs and are associated with few adverse effects [14]. Gepants inhibit CGRP mediated vasodilation but not result in vasoconstriction [32]. As such, they appear to be a safe alternative for patients in whom there are cardiovascular or vascular contraindications to the use of triptans. The most common treatment-emergent adverse effect is nausea for rimegepant [24]. In a meta-analysis, no significant adverse effects were observed with rimegepant compared to placebo [33]. The treatment associated side effects for atogepant are nausea, constipation and fatigue and liver-toxicity was not observed in doses up to 120 mg/day [34].

Even though, there is currently no evidence for the use of gepants in patients unresponsive to CGRP mAbs, in a recent phase 4 study rimegepant was shown to be non-inferior to galcanezumab in the preventive treatment of episodic migraine [30]. Moreover, the shorter half-life of gepants could be an advantage of gepants over CGRP mAbs in patients planning pregnancy. Low relative infant dose of rimegepant was shown in milk during the lactation period in healthy women suggesting that rimegepant could be a safe option during lactation [31] however, more studies are required. Both mAbs and gepants are effective treatments in migraine prevention and have safety and tolerability advantages, however, they have high cost burdens which still limit their use.

## “Less is more”

Of course clinical decisions in migraine, a complex and changing disease in the various phases of the life of the human being who suffers from it, are dictated by adherence to guidelines, but never detached from sound clinical experience [35–37]. Indeed, we know that the patient's therapeutic response is influenced by complex variables that are intrinsic to the patient or environmental in nature, and that the partial or non-response to a drug cannot be simplistically resolved by overlapping numerically more than one, especially if it has the same molecular target [38–40]. And here again the concept of *Less is more* seems entirely appropriate [41, 42].

## Data Availability

Not applicable.

## References

[1] Edvinsson L (2022). Calcitonin gene-related peptide (CGRP) is a key molecule released in acute migraine attacks-successful translation of basic science to clinical practice. J Intern Med.

[2] Russo AF, Hay DL. CGRP physiology, pharmacology, and therapeutic targets: migraine and beyond. Physiol Rev. 2023;103(2):1565–644. 10.1152/physrev.00059.2021 PMC9988538 36454715

[3] Sacco S, Amin FM, Ashina M, Bendtsen L, Deligianni CI, Gil-Gouveia R, Katsarava Z, MaassenVanDenBrink A, Martelletti P, Mitsikostas DD, Ornello R, Reuter U, Sanchez-Del-Rio M, Sinclair AJ, Terwindt G, Uluduz D, Versijpt J, Lampl C (2022). European Headache Federation guideline on the use of monoclonal antibodies targeting the calcitonin gene related peptide pathway for migraine prevention – 2022 update. J Headache Pain.

[4] Fiedler-Kelly J, Passarell J, Ludwig E (2020). Effect of fremanezumab monthly and quarterly doses on efficacy responses. Headache.

[5] Ashina M (2020). Migraine. N Engl J Med.

[6] Raffaelli B, Fitzek M, Overeem LH, Storch E, Terhart M, Reuter U (2023). Clinical evaluation of super-responders vs. non-responders to CGRP(-receptor) monoclonal antibodies: a real-world experience. J Headache Pain.

[7] Iannone LF, Fattori D, Marangoni M, Benemei S, Chiarugi A, Geppetti P, De Cesaris F (2023). Switching OnabotulinumtoxinA to monoclonal anti-CGRP antibodies in drug-resistant chronic migraine. CNS Drugs.

[8] Torres-Ferrús M, Gallardo VJ, Alpuente A, Caronna E, Gine-Cipres E, Pozo-Rosich P (2021). The impact of anti-CGRP monoclonal antibodies in resistant migraine patients: a real-world evidence observational study. J Neurol.

[9] Vandervorst F, Van Deun L, Van Dycke A, Paemeleire K, Reuter U, Schoenen J, Versijpt J (2021). CGRP monoclonal antibodies in migraine: an efficacy and tolerability comparison with standard prophylactic drugs. J Headache Pain.

[10] Barbanti P, Egeo G, Aurilia C, Altamura C, d’Onofrio F, Finocchi C, Albanese M, Aguggia M, Rao R, Zucco M, Frediani F, Filippi M, Messina R, Cevoli S, Carnevale A, Fiorentini G, Messina S, Bono F, Torelli P, Proietti S, Bonassi S, Vernieri F (2022). Italian migraine registry study group. Predictors of response to anti-CGRP monoclonal antibodies: a 24-week, multicenter, prospective study on 864 migraine patients. J Headache Pain.

[11] Al-Hassany L, Lyons HS, Boucherie DM, Farham F, Lange KS, Marschollek K, Onan D, Pensato U, Storch E, Torrente A, Waliszewska-Prosół M, Reuter U (2023). European Headache Federation School of Advanced Studies (EHF-SAS). The sense of stopping migraine prophylaxis. J Headache Pain.

[12] Barbanti P, Aurilia C, Egeo G, Torelli P, Proietti S, Cevoli S, Bonassi S (2023). Italian Migraine Registry study group. Late response to Anti-CGRP monoclonal antibodies in migraine: a multicenter prospective observational study. Neurology.

[13] di Schiano F, Bolchini M, Ceccardi G, Caratozzolo S, Liberini P, Rao R, Padovani A (2023). An observational study on monoclonal antibodies against calcitonin-gene-related peptide and its receptor. Eur J Neurol.

[14] Lampl C, MaassenVanDenBrink A, Deligianni CI, Gil-Gouveia R, Jassal T, Sanchez-Del-Rio M, Reuter U, Uluduz D, Versijpt J, Zeraatkar D, Sacco S (2023). The comparative effectiveness of migraine preventive drugs: a systematic review and network meta-analysis. J Headache Pain.

[15] Ziegeler C, May A (2020). Non-responders to treatment with antibodies to the CGRP-receptor may profit from a switch of antibody class. Headache.

[16] Straube A, Broessner G, Gaul C (2023). Real-world effectiveness of fremanezumab in patients with migraine switching from another mAb targeting the CGRP pathway: a subgroup analysis of the finesse study. J Headache Pain.

[17] Ziegeler C, Mehnert J (2020). Central effects of erenumab in migraine patients: an event-related functional imaging study. Neurology.

[18] Basedau H, Sturm LM (2022). Migraine monoclonal antibodies against CGRP change brain activity depending on ligand or receptor target - an fMRI study. Elife.

[19] Hong JB, Lange KS, Overeem LH, Triller P, Raffaelli B, Reuter U (2023). A scoping review and meta-analysis of anti-CGRP monoclonal antibodies: predicting response. Pharmaceuticals (Basel).

[20] Nowaczewska M, Straburzyński M, Waliszewska-Prosół M, Meder G, Janiak-Kiszka J, Kaźmierczak W (2022). Cerebral blood flow and other predictors of responsiveness to erenumab and fremanezumab in migraine-a real-life study. Front Neurol.

[21] Yuan H, Spare NM, Silberstein SD (2019). Targeting CGRP for the prevention of migraine and cluster headache: a narrative review. Headache.

[22] Moreno-Ajona D, Pérez-Rodríguez A, Goadsby PJ (2020). Gepants, calcitonin-gene-related peptide receptor antagonists: what could be their role in migraine treatment?. Curr Opin Neurol.

[23] Lipton RB, Croop R, Stock DA, Madonia J, Forshaw M, Lovegren M (2023). Safety, tolerability, and efficacy of zavegepant 10 mg nasal spray for the acute treatment of migraine in the USA: a phase 3, double-blind, randomised, placebo-controlled multicentre trial. Lancet Neurol.

[24] Croop R, Lipton RB, Kudrow D, Stock DA, Kamen L, Conway CM (2021). Oral rimegepant for preventive treatment of migraine: a phase 2/3, randomised, double-blind, placebo-controlled trial. Lancet.

[25] Ailani J, Lipton RB, Goadsby PJ, Guo H, Miceli R, Severt L, Finnegan M, Trugman JM, ADVANCE Study Group (2021). Atogepant for the preventive treatment of migraine. N Engl J Med.

[26] Pan KS, Siow A, Hay DL, Walker CS (2020). Antagonism of CGRP signaling by rimegepant at two receptors. Front Pharmacol.

[27] Goadsby PJ, DD, Trugman JM, Finnegan M, Lakkis H, Lu K, et al (2019) Orally Administered Atogepant Was Efficacious, Safe, and Tolerable for the Prevention of Migraine: Results From a Phase 2b/3 Study 92(15 Supplement):S17.001

[28] Luo G, Chen L, Conway CM, Denton R, Keavy D, Signor L (2012). Discovery of (5S,6S,9R)-5-amino-6-(2,3-difluorophenyl)-6,7,8,9-tetrahydro-5H-cyclohepta[b]pyridin-9-yl 4-(2-oxo-2,3-dihydro-1H-imidazo[4,5-b]pyridin-1-yl)piperidine-1-carboxylate (BMS-927711): an oral calcitonin gene-related peptide (CGRP) antagonist in clinical trials for treating migraine. J Med Chem.

[29] Moreno-Ajona D, Villar-Martínez MD, Goadsby PJ (2022). New generation gepants: migraine acute and preventive medications. J Clin Med.

[30] Schwedt TJ, Myers Oakes TM, Martinez JM, Vargas BB, Pandey H, Pearlman EM, Richardson DR, Varnado OJ, Cobas Meyer M, Goadsby PJ (2023) Comparing the efficacy and safety of galcanezumab versus rimegepant for prevention of episodic migraine: results from a randomized. Controlled Clinical Trial Neurol Ther. 10.1007/s40120-023-00562-w. (Epub ahead of print. PMID: 37948006) 10.1007/s40120-023-00562-w PMC10787669 37948006

[31] Baker TE, Croop R, Kamen L, Price P, Stock DA, Ivans A, Bhardwaj R, Anderson MS, Madonia J, Stringfellow J, Bertz R, Coric V, Hale TW (2022). Human milk and plasma pharmacokinetics of single-dose rimegepant 75 mg in healthy lactating women. Breastfeed Med.

[32] Schwedt TJ, Lipton RB, Ailani J, Silberstein SD, Tassorelli C, Guo H, Lu K, Dabruzzo B, Miceli R, Severt L, Finnegan M, Trugman JM (2022). Time course of efficacy of atogepant for the preventive treatment of migraine: results from the randomized, double-blind ADVANCE trial. Cephalalgia.

[33] Gao B, Yang Y, Wang Z, Sun Y, Chen Z, Zhu Y, Wang Z (2020). Efficacy and safety of rimegepant for the acute treatment of migraine: evidence from randomized controlled trials. Front Pharmacol.

[34] Goadsby PJ, Dodick DW, Ailani J, Trugman JM, Finnegan M, Lu K, Szegedi A (2020). Safety, tolerability, and efficacy of orally administered atogepant for the prevention of episodic migraine in adults: a double-blind, randomized phase 2b/3 trial. Lancet Neurol.

[35] Pohl H, Gantenbein AR, Sandor PS (2020). A survey on probable and improbable decisions about headache treatment. SN Compr. Clin Med.

[36] Singh A, Gupta D, Sahoo AK (2020). Acute migraine: can the new drugs clinically outpace? SN Compr. Clin Med.

[37] Schoenen J, Van Dycke A, Versijpt J, Paemeleire K (2023). Ten open questions in migraine prophylaxis with monoclonal antibodies blocking the calcitonin-gene related peptide pathway: a narrative review. J Headache Pain.

[38] Shah T, Bedrin K, Tinsley A (2023). Calcitonin gene relating peptide inhibitors in combination for migraine treatment: a mini-review. Front Pain Res (Lausanne).

[39] Holzer P, Holzer-Petsche U (2022). Constipation caused by anti-calcitonin gene-related peptide migraine therapeutics explained by antagonism of calcitonin gene-related peptide’s motor-stimulating and prosecretory function in the intestine. Front Physiol.

[40] Joshi N, McAree M, Klimowich K (2020). Oral candidiasis in a migraine patient taking erenumab and galcanezumab: a case report. SN Compr Clin Med.

[41] Martelletti P (2020). Combination therapy in migraine: asset or issue?. Expert Rev Neurother.

[42] Martelletti P, Luciani M, Spuntarelli V, Bentivegna E (2021). Deprescribing in migraine. Expert Opin Drug Saf.

